# Kinetics of Abnormal Prion Protein in Blood of Transgenic Mice Experimentally Infected by Multiple Routes with the Agent of Variant Creutzfeldt–Jakob Disease

**DOI:** 10.3390/v15071466

**Published:** 2023-06-28

**Authors:** Oksana Yakovleva, Teresa Pilant, David M. Asher, Luisa Gregori

**Affiliations:** U.S. Food and Drug Administration, Center for Biologics Evaluation and Research, Silver Spring, MD 20993, USA; oksana.yakovleva@fda.hhs.gov (O.Y.); teresa.pilant@fda.hhs.gov (T.P.) davidmasher@starpower.net (D.M.A.)

**Keywords:** prion, macaque, mouse, blood, detection

## Abstract

Transmissible spongiform encephalopathies (TSEs) or prion diseases are characterized by the accumulation in affected tissues of the abnormal prion protein PrP^TSE^. We previously demonstrated PrP^TSE^ in the blood of macaques experimentally infected with variant Creutzfeldt–Jakob disease (vCJD), a human TSE, months to years prior to clinical onset. That work supported the prospect of using PrP^TSE^ as a blood biomarker to detect vCJD and possibly other human TSEs before the onset of overt illness. However, our results also raised questions about the origin of PrP^TSE^ detected in blood early after inoculation and the effects of dose and route on the timing of the appearance of PrP^TSE^. To investigate these questions, we inoculated vCJD-susceptible transgenic mice and non-infectable prion protein-knockout mice under inoculation conditions resembling those used in macaques, with additional controls. We assayed PrP^TSE^ in mouse blood using the protein misfolding cyclic amplification (PMCA) method. PrP^TSE^ from the inoculum cleared from the blood of all mice before 2 months post-inoculation (mpi). Mouse PrP^TSE^ generated de novo appeared in blood after 2 mpi. These results were consistent regardless of dose or inoculation route. We also demonstrated that a commercial ELISA-like PrP^TSE^ test detected and quantified PMCA products and provided a useful alternative to Western blots.

## 1. Introduction

Transmissible spongiform encephalopathies (TSEs or prion diseases) are rare, fatal neurodegenerative disorders of animals and humans. TSEs are characterized by the accumulation in affected tissues of the abnormally folded relatively protease-resistant prion protein named PrP^TSE^. TSEs have long asymptomatic incubation periods during which infected individuals might donate potentially contaminated biological materials such as blood, tissues, and cells for therapeutic purposes [1]. Variant Creutzfeldt–Jakob disease (vCJD) is a human TSE most often acquired from consumption of food contaminated with the agent that causes bovine spongiform encephalopathy (BSE) in cattle [2,3,4]. Measures to stop the spread of BSE and vCJD were implemented worldwide, and as a result, very few cases of BSE and no new cases of vCJD have been reported in the past few years [5,6]. Among other human TSEs, sporadic CJD (sCJD) is the most common; familial CJD (fCJD) is extremely rare. Family members with certain mutations in the prion protein-encoding (*PRNP*) gene have an increased risk of developing CJD [7,8].

PrP^TSE^ is currently the only known TSE biomarker; thus, it is the target for all biochemical diagnostic tests under development. The most promising PrP^TSE^ detection methods are protein misfolding cyclic amplification (PMCA) [9] and real-time quaking-induced conversion (RT-QuIC) [10]. Both are in vitro methods that amplify PrP^TSE^ molecules and allow their detection even when PrP^TSE^ is present in extremely low levels such as in biological samples [9,10,11]. PMCA amplified PrP^TSE^ from blood of individuals with vCJD [12,13,14]. In another study, PrP^TSE^ in the blood of vCJD patients was detected using a selective capture method without amplification [15]. Detection of PrP^TSE^, and infectivity, has yielded inconsistent results in various studies with sCJD blood [13,14,16]. Although new biomarkers and diagnostic tests have been developed to improve CJD diagnostics [7], the field still lacks a reliable, rapid, specific, and sensitive blood-based test to identify humans with TSEs during the preclinical phase of infection. Early detection of PrP^TSE^ would benefit individuals with sCJD and members of families with a history of fCJD by allowing therapeutic trials to be initiated before neurodegeneration becomes severe and irreversible. An assay that identifies persons incubating sCJD might also be useful to screen living donors of human cellular and tissue-derived products, reducing the risk of iatrogenic CJD [1,7].

Several years ago, we began a study to develop a method to detect PrP^TSE^ in the blood of vCJD-infected macaques as a prototype diagnostic test for human vCJD and possibly sCJD [17]. The animals were infected peripherally (intravenous (IV) and intraperitoneal (IP) administration). We generated longitudinal panels of infected animal blood used to track the appearance of PrP^TSE^ over time correlated with the onset of overt (“clinical”) illness [18]. PrP^TSE^, detected by PMCA, appeared in the blood of cynomolgus macaques as early as 2 months post-inoculation (2 mpi)—the earliest time tested. This unexpected early detection of PrP^TSE^ occurred long before the onset of clinical illness in macaques: 25.5 months for two macaques and 21 months for one macaque before clinical onset [18]. When we conducted a similar study with rhesus macaques injected intraperitoneally with 10 times less vCJD agent compared to the dose used with cynomolgus macaques, PrP^TSE^ appeared in blood 5 and 11 months before clinical onset in two animals, while PrP^TSE^ was not reproducibly detected in one rhesus until 1 month after clinical signs had begun [18]. Although these results confirmed and supported the possible use of PrP^TSE^ as a future preclinical diagnostic biomarker in blood, they also left some unanswered questions that required further investigations. Specifically, we needed to establish whether PrP^TSE^ detected 2 mpi was truly generated de novo as a response to infection or was simply recovered from the residual circulating inoculum. Next, we wanted to assess possible causes of the dramatic difference in the time of appearance of detectable PrP^TSE^ in the blood of rhesus and cynomolgus macaques. To address this latter goal, we focused on the different doses of inoculum injected into the two animal species.

Those basic questions were more feasibly addressed using mice instead of macaques. We used TgBo110 transgenic mice [19] because they are highly susceptible to infection with the vCJD agent and have been used extensively by us and others to study BSE and vCJD [18,19,20,21]. We also used PrP-knockout mice resistant to TSE infections [22] to track clearance of the inoculum without the confounding effects of PrP^TSE^ produced by the infected mice. We attempted to mimic the conditions we previously used to infect macaques with the goal of determining when mouse-generated PrP^TSE^ first appeared in blood and whether the time of appearance was delayed in mice inoculated under conditions matching those used to infect rhesus macaques. Previous studies detected PrP^TSE^ in the blood of rodents experimentally infected with TSEs [23,24], but those studies did not account for PrP^TSE^ remaining from the inoculum. Our studies addressed this consideration. We also explored how the route of inoculation (IV, IP, and intracerebral (IC)—the most commonly studied routes of experimental TSE infection) affected the time when PrP^TSE^ was first detected in blood. Furthermore, we introduced a rapid method to detect PrP^TSE^ products generated by PMCA that could, in some cases, substitute for the Western immunoblots, simplifying test readout and improving throughput. We believe that our data will contribute to the future development and validation of TSE assays based on PrP^TSE^ detection in blood.

## 2. Materials and Methods

### 2.1. Mouse Models and Inoculations

TgBo110 mice overexpressing normal bovine prion protein (called “cellular” PrP or PrP^C^) approximately 8-fold compared to PrP^C^ in brains of cows [19] were originally derived at the Centro de Investigación en Sanidad Animal, Instituto Nacional de Investigación y Tecnología Agraria y Alimentaria (CISA-INIA, Madrid, Spain). PrP-KO mice FVB Prnp−/− on FVB/NJ genetic background were kindly donated by Jaroslav Vostal [25] (U.S. Food and Drug Administration, White Oak, Silver Spring) MD, USA. Both mouse strains were bred at the U.S. Food and Drug Administration and confirmed by PCR to have the expected genotypes.

To reduce the volumes of inocula injected into mice to match the inoculation conditions previously used to infect cynomolgus and rhesus macaques [18], we used ratios of circulating blood volumes of macaques and mice. The average volume of circulating blood in adult cynomolgus and rhesus macaques is about 60 mL/kg [26], and the average weight of our macaques at the time of inoculation was 7.5 kg, yielding an estimated average of 450 mL of blood in a macaque. The average volume of circulating blood in a young adult mouse is 72 mL/kg [26], and our mice weighed on average 26 g at inoculation; thus, the average volume of circulating blood in our mice was estimated to be about 1.8 mL. Therefore, we used a 250:1 ratio to calculate appropriate inoculation doses for mice for all inoculation routes. We inoculated groups of 6 to 10 TgBo110 mice and groups of 10 PrP-KO mice by 3 different routes (IV, IP, and IC) and a combination of routes with a homogenate of brain tissue from a cynomolgus macaque with histopathologically confirmed vCJD. We inoculated mice intracerebrally with 30 µL of 1% brain homogenate [18,20]. The 6 inoculation conditions tested and the volumes of inoculum injected are shown in Table 1.

### 2.2. Blood and Tissue Collections

We collected blood 1 week after inoculation of TgBo110 mice and then every month post-inoculation (mpi) for 2 years until the mice were euthanized. We drew blood from PrP-KO mice using the same schedule but terminated collections at 7 mpi because these mice were not expected to develop TSE. We collected blood by cutting the submandibular vein, obtaining 60–100 µL of citrated blood from each mouse at each time point; we pooled the blood from mice in each inoculation group to obtain sufficient volumes of sample to conduct 2 PMCA tests and immediately stored aliquots of pooled blood at −80 °C. Mice were euthanized when signs such as loss of body weight, scruffy hair coat, and lethargy were observed or when requested by veterinary staff for reasons of animal welfare. We collected approximately 1 mL of citrated blood from each mouse (not pooled) by cardiac puncture before euthanasia and necropsy. The brain and spleen were harvested at necropsy and frozen for further testing. Brain tissue to detect PrP^TSE^ was always excised from the hemisphere opposite the site of injection. Blood, spleens, and brains were assayed for PrP^TSE^ by PMCA. 

### 2.3. Detection of PrP^TSE^ in Mouse Blood by PMCA

As previously described [18], we tested 250 µL aliquots of citrated whole blood treated with a final concentration of 20 U/mL of benzonase nuclease (Millipore-Sigma, Burlington MA, USA) for 30 min at room temperature with constant mixing. Next, we added 250 µL of 20% molecular-grade Sarkosyl (Sigma-Aldrich, St. Louis, MO, USA) and incubated the mixture at room temperature for 30 min with mixing. We ultracentrifuged samples (1 h at 100,000× *g*, 10 °C), discarded supernatants, and resuspended pellets in 100 µL of a 10% homogenate (PMCA “substrate”) of brains of red-backed voles (bred in-house) [27] in buffer, 50 mL of which contained 0.5 g laboratory-grade Triton X100 (Sigma-Aldrich, St. Louis, MO, USA), 1.5 mL of 5 M molecular-grade NaCl, and 48 mL PBS without calcium and magnesium, pH 7.2; 1 protease inhibitor cocktail tablet containing EDTA (Roche, Sigma-Aldrich, St. Louis, MO, USA) was added immediately prior to use. We transferred the resuspended pellets to 0.2 mL tubes containing three 3/32 in. PTFE beads (McMaster-CARR, Elmhurst, IL, USA) and conducted PMCA using a titanium cuphorn in a programmable Misonix Q700 sonicator (QSonica, Newtown, CT, USA) filled with water and kept inside an incubator set at 37 °C. Each cycle of sonication comprised 10 s pulses every 15 min. The first round of sonication lasted a total of 72 h. After the first round of sonication, we diluted samples 1:10 with fresh vole brain homogenate substrate to conduct round 2 of PMCA and repeated the procedure for round 3; rounds 2 and 3 each lasted 24 h.

To detect PrP^TSE^ in PMCA products by Western blot, we treated aliquots of samples after PMCA, with 50 µg/mL proteinase K for 1 h at 37 °C to remove normal prion protein and denaturing NuPAGE LDS sample buffer (4×) with NuPAGE sample-reducing agent heated at 70 °C for 10 min; we then separated proteins using SDS-PAGE on 12% Bis-Tris precast gels (Invitrogen-Thermo Fisher Scientific, Waltham, MA, USA). We detected prion protein transferred to PVDF membranes using mouse anti-PrP monoclonal antibody 6D11 at a concentration of 6 mg/mL (Research Foundation for Mental Hygiene, New York State Institute for Basic Research) diluted 1:5000 and an HRP-conjugated secondary antibody and then captured chemiluminescent signals on the membranes using the Bio-Rad ChemiDoc Imaging System (Bio-Rad, Hercules, CA, USA).

### 2.4. Detection of PMCA-Generated Products Using a Rapid PrP^TSE^ Immunoassay

We measured levels of PMCA products using a colorimetric enzyme-linked immunosorbent assay (ELISA-like) called IDEXX HerdChek BSE-Scrapie Antigen Test kit (HC PrP^TSE^ test) that detects PrP^TSE^ as previously described [18]. For this assay, we used 25 µL aliquots of samples collected after rounds 2 and 3 of PMCA, 25 µL of working buffer from the assay kit, and PBS to a total volume of 100 µL. We performed HC assays according to the manufacturer’s instructions (IDEXX Laboratories, Westbrook, ME, USA). We analyzed only rounds 2 and 3 because PrP^TSE^ was undetectable by both Western blot and HC tests after the first round of PMCA.

### 2.5. Detection of PrP^TSE^ in Mouse Spleen and Brain by PMCA

We gently homogenized 50 mg samples of thawed spleen tissue using sterile disposable pestles in 0.5 mL of PBS. Unhomogenized tissue was discarded, and the suspension was treated with 50 U/mL of benzonase nuclease for 30 min at room temperature with constant agitation. Next, we added 0.5 mL of 20% molecular-grade Sarkosyl to each sample and incubated it at room temperature for 30 min with agitation. We performed ultracentrifugation, PMCA, and Western blot analyses as described above for mouse blood.

We homogenized 10% mouse brain in PBS using a Mini Beadbeater (Biospec Products, Bartlesville, OK, USA) twice for 1.5 min followed by cooling for 5 min on ice. We tested each mouse brain for PrP^TSE^ using PMCA with 25 µL of 10% brain homogenate in 75 µL of normal vole brain homogenate. PMCA and Western blots were performed as described above.

## 3. Results

### 3.1. Mouse Inoculations

We inoculated six groups of TgBo110 mice under conditions indicated in Table 1 and further described in Figure S1 in Supplementary Materials. The first two conditions resembled those used to inoculate cynomolgus and rhesus macaques, respectively. Notably, the only difference in inoculations of the two macaque species was that rhesus macaques received 10 times less inoculum by the IP route (IP-low) compared to that injected into cynomolgus macaques by that route (IP-high). IV inoculations were the same for all macaques. We inoculated mice by the IC route—a route not used to inject macaques—to serve as a positive control since we knew from previous studies that IC inoculation of this material at the dilution used should infect all TgBo110 mice. To complete the study, we also injected TgBo110 mice by either the IV or IP route separately. The latter route was also tested at two concentrations of brain suspension, high and low, to match the different concentrations used to inoculate cynomolgus and rhesus macaques intraperitoneally. We repeated the same six conditions of inoculation with PrP-KO mice, as described in Table 1; PrP-KO mice do not develop vCJD, and thus, any PrP^TSE^ detected in their blood must have come from the inoculum, allowing us to determine the kinetics of clearance of PrP^TSE^ remaining from the inoculum after each condition of inoculation.

### 3.2. PMCA Products Detected Using ELISA-like Assay

**Figure 1:**
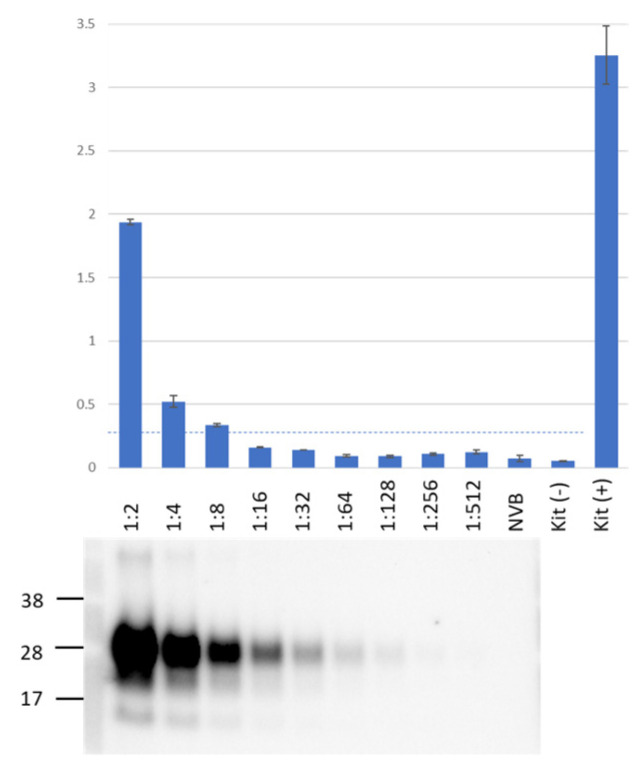
Direct comparison of assay sensitivity for HerdChek (HC) PrP^TSE^ test and Western blot. PMCA products were serially diluted 2-fold in 10% normal vole brain. We used 25 µL of each sample for HC PrP^TSE^ tests. After proteinase K digestion, the equivalent of 10 µL of each sample was assayed by Western blot. The top panel also shows colorimetric results for each sample and for internal controls (negative and positive) included in the HC kit. The horizontal dotted line corresponds to the assay threshold (O.D. 0.31) [28]. The Western blot lanes were aligned with corresponding HC samples. NVB, normal vole brain (PMCA substrate). Representative results from a total of three independent experiments.

We used PMCA to test all blood samples collected during the study, using Western blot to confirm the presence of PMCA products. To detect those products, we employed an ELISA-like assay: a commercial HC PrP^TSE^ test based on a proprietary ligand that selectively binds and concentrates aggregated PrP^TSE^ but not monomeric PrP, requiring no preliminary protease treatment of samples to remove normal PrP^C^ (i.e., no proteinase K digestion) as required to detect PrP^TSE^ by Western blot. We directly compared the sensitivity of the HC PrP^TSE^ test to that of the Western blot (Figure 1) using 2-fold serial dilutions of a PMCA product. PrP^TSE^ signal was detected by Western blot in samples diluted 1:128; the HC PrP^TSE^ test detected PrP^TSE^ only in samples diluted ≤ 1:8, the lowest dilution at which PrP^TSE^ was consistently detected above a threshold value of 0.31 O.D. at 562 nm [27]. This value was established as 3 times the standard deviation of average O.D. values of negative human brain homogenates [27].

These results contrast with our previous data showing that the HC PrP^TSE^ test detected PrP^TSE^ in human sCJD brain homogenates with approximately 30-fold higher sensitivity than that of Western blot [28]. This finding might be explained if the HC PrP^TSE^ test detected human PrP^TSE^ better than vole PrP^TSE^ (PMCA products in this study were generated using vole brain as the normal PrP substrate) or if PrP^TSE^ in our PMCA products had a conformation that did not bind to the ligand as well as natural PrP^TSE^, reducing its detection by HC test. Despite this limitation, the HC PrP^TSE^ test was useful to compare PMCA signals from the blood and tissues of inoculated mice.

### 3.3. PrP^TSE^ in the Blood of PrP-KO Mice

**Figure 2:**
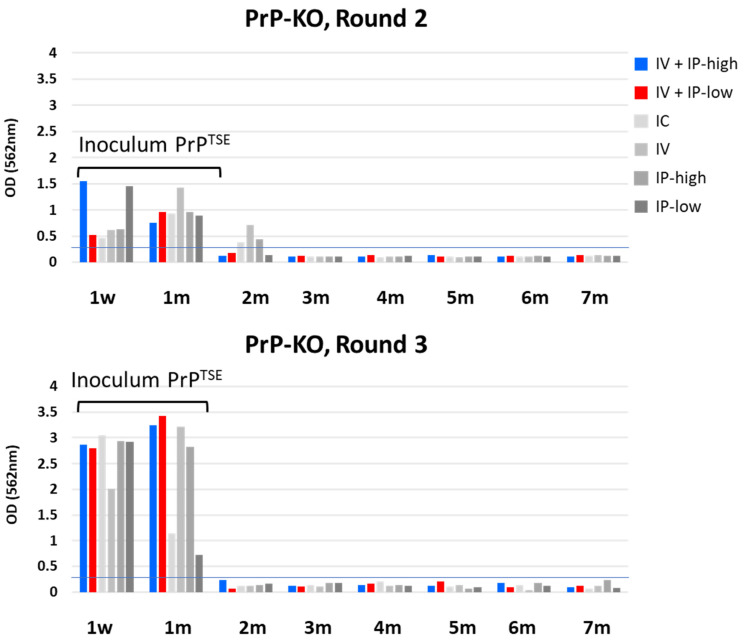
HC PrP^TSE^ test results for PrP-KO mice. Six groups of PrP-KO mice were inoculated as indicated in the figure. Importantly, “IV + IP-high” matched inoculation conditions used to inoculate cynomolgus macaques, and “IV + IP-low” matched conditions used to inoculate rhesus macaques [18]. Blood aliquots from inoculated mice collected at the indicated times were tested for PrP^TSE^ by PMCA. PMCA products were assayed by HC PrP^TSE^ test, and results for rounds 2 (top panel) and 3 (lower panel) are reported as the colorimetric signal versus collection time. w, week; m, month; IV, intravenous; IP, intraperitoneal; IC, intracerebral.

Figure 2 shows HC PrP^TSE^ test results after PMCA of blood from PrP-KO mice inoculated under the six experimental conditions (Table 1). The first two columns model the inoculation conditions used to infect cynomolgus and rhesus macaques.

**Figure 3:**
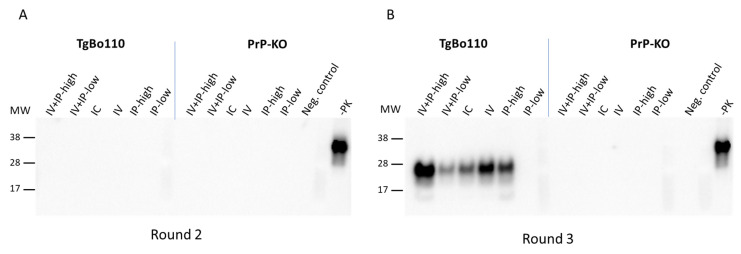
Comparison of blood of vCJD-inoculated TgBo110 and PrP-KO mice collected 2 months post-inoculation after rounds 2 and 3 of PMCA. Blood aliquots of TgBo110 and PrP-KO mice inoculated as indicated in the figure were assayed for PrP^TSE^ using PMCA. PMCA products were visualized after rounds 2 (panel (**A**)) and 3 rounds (panel (**B**)), after proteinase K digestion, on Western blots using antibodies against PrP. “Neg. control”, normal vole brain substrate after PMCA and treatment with proteinase K; “-PK”, normal vole brain without PMCA and not treated with proteinase K; MW, molecular weight markers.

We detected PMCA products at 1 week and 1 mpi in rounds 2 and 3 of PMCA in blood samples from mice inoculated under all conditions (Figure 2). As expected, the overall levels of PMCA products were higher after round 3 than after round 2 because concentrations of PrP^TSE^ increased with each successive cycle of sonication. PrP^TSE^ was also detected above the threshold in some blood samples after round 2, but no signals were present in PMCA products collected after round 3. Upon close review of Western blots, we detected no PrP^TSE^ in those same samples after PMCA rounds 2 and 3 (Figure 3: PrP-KO blots in panels A and B). Based on these observations, we concluded that the reactive signals detected 2 mpi were probably artifacts of the HC PrP^TSE^ test; we do not know the origin of these apparent false positive signals.

### 3.4. PrP^TSE^ in the Blood of TgBo110 Mice

**Figure 4:**
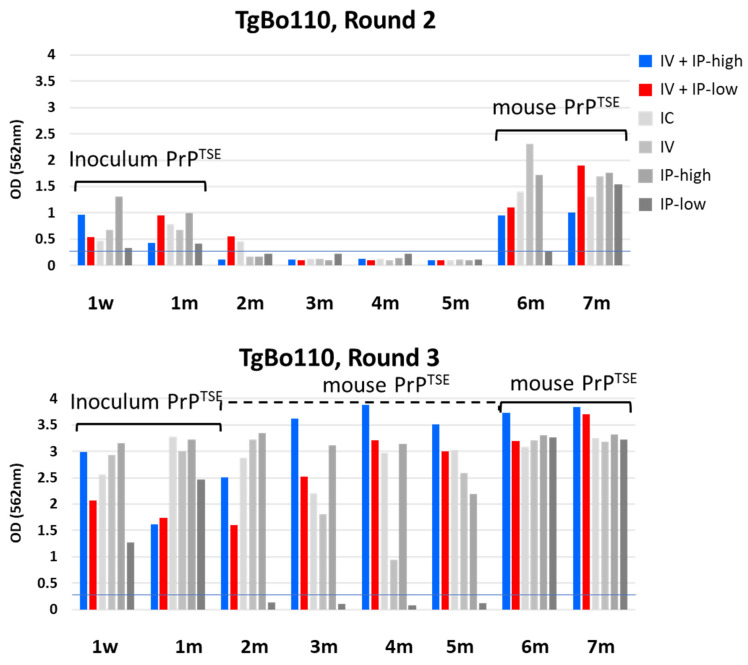
HC PrP^TSE^ test results for TgBo110 mice. Six groups of TgBo110 mice were inoculated as indicated in the figure. Importantly, the “IV + IP-high” condition matched inoculation conditions for cynomolgus macaques, and “IV + IP-low” matched inoculation conditions for rhesus macaques, as previously reported [18]. Blood aliquots from inoculated mice collected at the indicated times were tested for PrP^TSE^ by PMCA. PMCA products were assayed by HC PrP^TSE^ test, and results for rounds 2 (top panel) and 3 (lower panel) are reported as the colorimetric signal versus collection time. w, week; m, month; IV, intravenous; IP, intraperitoneal; IC, intracerebral.

We detected PrP^TSE^ remaining from the inoculum in the blood of TgBo110 mice (Figure 4) up to 1 mpi, a finding consistent with those seen with PrP-KO mice (Figure 2). As previously noted, we observed weak signals by HC testing in blood samples from some mice 2 mpi, not confirmed to be PrP^TSE^ by Western blot (Figure 3: TgBo110 blot panel A).

Once PrP^TSE^ from the inoculum had cleared, no PrP^TSE^ was detected by PMCA during the next 4 months. PrP^TSE^ again appeared 6 mpi in the blood of mice inoculated with vCJD agent under all conditions except not in blood from mice inoculated under IP-low conditions. Seven months post-inoculation, similar amounts of PrP^TSE^ amplified by PMCA were detected in the blood of mice inoculated under all conditions. We conclude that this second wave of PrP^TSE^ detected by PMCA must have been generated de novo by infected mice. Interestingly, round 3 of PMCA detected PrP^TSE^ even in samples negative in round 2. We confirmed these results by Western blot (Figure 3B, left panel) showing PrP^TSE^ in the blood of mice inoculated under all conditions except for the IP-low condition. These observations indicated that de novo mouse-generated PrP^TSE^ was already present in the blood of TgBo110 mice as early as 2 to 5 mpi, but only in very low concentrations—undetectable by the first two rounds of PMCA. It is also important to note that the results for mice inoculated under two experimental conditions mimicking those previously used with cynomolgus and rhesus macaques showed similar results. These data suggest that the difference in dose of inoculum did not affect the time of appearance of PrP^TSE^ in blood.

### 3.5. Final Disease Status of Each Mouse in the Study

We monitored TgBo110 mice until the end of the study and confirmed that de novo-generated PrP^TSE^ remained detectable in their blood from its first appearance until terminal illness. Table 2 shows a complete analysis of TgBo110 mice with results from PMCA tests of blood, brain, and spleen for each animal. All mice, except five, at the time of death, had evidence of infection confirmed by PMCA reactivity in at least one tissue tested: blood, brain, and spleen. Of the five mice with negative results, two died too early to know whether they were infected; the brains of two other mice were negative, but their blood and spleen—earlier indicators of infection—could not be tested. One mouse in the IP-low cohort was truly uninfected because it died late in the vCJD incubation period and the PMCA of all three tissues was negative. Scheduled euthanasia was conducted 4 mpi (for one mouse 3 mpi) to determine the status of TgBo110 mice at early time points when we expected animals to have no indications of vCJD. In tissues of mice inoculated under all conditions except IC inoculations, blood, and spleen were PMCA-positive before PrP^TSE^ was detected in the brain. To our surprise, PrP^TSE^ was already detectable in the blood (and spleen) as early as 4 mpi except for IP-low conditions. It is also noteworthy that PrP^TSE^ was detectable in the brain no earlier than 12 mpi while blood was PrP^TSE^-positive already at 3 or 4 mpi for mice inoculated under all conditions except for mice inoculated under the IP-low condition. These results confirm that, regardless of the inoculation route, blood and spleen are affected very early in the infection. When we compared mice inoculated under conditions intended to model previous cynomolgus and rhesus inoculations, we observed that the blood of both cohorts of mice became PrP^TSE^-positive at the same times, as early as 3 or 4 mpi.

## 4. Discussion

PrP^TSE^ is currently the only biomarker specific for TSE diseases. However, we know very little about this protein, especially regarding the properties of PrP^TSE^ in blood: its biochemical characteristics, its time of appearance in preclinical phases of infection and clinical illness, and changes in its concentration during the course of infection and from individual to individual. These are important questions to address before accepting PrP^TSE^ as a reliable biomarker suitable to develop into useful antemortem diagnostic assays for human TSEs. Studying PrP^TSE^ in blood poses challenges, the first being due to its very low concentration requiring in vitro PrP^TSE^ amplification tests such as PMCA or RT-QuIC for its detection and the second being the lack of blood samples collected from infected humans spanning the period from preclinical “incubation period” to overt clinical illness. To overcome the second limitation, we and other investigators experimentally infected large TSE-susceptible animals and collected their blood throughout the incubation period for testing to detect PrP^TSE^. For example, PrP^TSE^ was first detected in the blood of transfused sheep 6 months post-exposure, the earliest time point tested, and then found continuously in blood samples collected before clinical onset and during overt illness until euthanasia [29]. In another study, longitudinal blood samples from macaques infected intravenously with brain-derived vCJD agent tested PrP^TSE^-positive by PMCA as early as 10 mpi [12]. RT-QuIC detected PrP^TSE^ in the blood of deer 1–2 days post-inoculation, after which the signal declined but subsequently increased sharply at 3 mpi, remaining constant for several months through the end of the study [30]. Those results are consistent with the findings of Soto and colleagues, and our own, using blood of macaques experimentally infected with vCJD by the IV and IP routes [18,31]. Collectively, these studies revealed that PrP^TSE^ could be detected in blood relatively soon after exposure and for several months before clinical onset. However, these studies left open the possibility that the PrP^TSE^ detected early was a residuum from the inoculum remaining in the blood. This option could not be excluded because the in vitro amplification assays did not distinguish PrP^TSE^ from the brain (the inoculum) or blood (generated by infected animals de novo). We used mice to investigate this question. Compared to nonhuman primates, mice have the advantage of being available as PrP-KO animals, unable to be infected with TSEs or to generate endogenous PrP^TSE^, allowing us to follow the fate of PrP^TSE^ remaining from the inoculum. Mice are also ideal for comparative studies using different routes of inoculation, research requiring an unfeasible number of nonhuman primates. Mice are inbred and homogeneous, unlike macaques which are outbred and more variable in genome and phenotype. Caveats regarding mouse studies are that PrP^TSE^ in their blood might be cleared by mechanisms that differ from those of primates, yielding differences in kinetics. Blood samples must also be pooled from several mice in the same cohort to obtain sufficient volumes for testing. Pooling blood samples loses the opportunity to investigate the variations from animal to animal that we observed with our studies in macaques.

Studies with PrP-KO mice informed us that PrP^TSE^ from the inoculum remained detectable up to 1 month post-exposure but was completely cleared by 2 mpi. These results were consistent for all conditions of inoculation tested. The same clearance of PrP^TSE^ originating from the inoculum was also observed with TgBo110 mice and followed by a period of 4 months during which extremely low levels of PrP^TSE^ in the blood required three rounds of PMCA to detect. PrP^TSE^ then increased dramatically in blood 6 mpi and remained constant throughout the rest of the incubation period and during overt vCJD. Because PrP^TSE^ was not detected in the blood of PrP-KO mice 2 mpi even by round 3 of PMCA, we can be confident that PrP^TSE^ was generated de novo by vCJD-infected TgBo110 mice. Mice infected under all conditions of inoculation showed the same trend of PrP^TSE^ detected in blood. Although PrP^TSE^ clearance in mice might be different than in macaques, the complete clearance of inoculated PrP^TSE^ from the blood of our PrP-KO mice during the first two months after inoculations suggests that the same could have occurred in infected macaques. If this is correct, PrP^TSE^ was released into the blood of infected cynomolgus macaques more than 2 years before they showed clinical signs of vCJD. This conclusion is consistent with reported cases of transfusion-transmitted vCJD in recipients of blood donated 18 to 40 months before donors became clinically ill with vCJD [6]. Our results are also in agreement with the detection of PrP^TSE^ in the blood of two vCJD-infected asymptomatic individuals 14 and 31 months prior to the earliest clinical signs of illness [14]. Taken together, these and our findings are encouraging for prospects to develop informative assays that detect PrP^TSE^ in the blood of infected people before the clinical onset of illness. In addition, our observations highlight the importance of beginning the collection of blood samples early when testing for PrP^TSE^ in future experimental studies of TSEs in large animals.

We showed that PrP^TSE^ from the inoculum remained circulating in the blood of inoculated mice for at least 1 month, a time that differs from a half-life of only a few hours estimated by a study with purified radiolabeled and non-radiolabeled PrP^TSE^ inoculated intravenously into mice [32]. There could be several reasons for these different results. An example is differences in the preparation of inocula: in our studies, PrP^TSE^ was in crude brain homogenate preparations also containing a variety of proteins, lipids, and other components that might protect PrP^TSE^ from degradation or retard its uptake into tissues. In contrast, radiolabeled studies used highly purified and chemically modified PrP^TSE^. Furthermore, we injected more PrP^TSE^, which may have prolonged the time PrP^TSE^ circulated in blood. Importantly, Urayama and colleagues, in the same studies, showed rapid uptake of IV-injected PrP^TSE^ by the spleen and other lymphoid tissues, sites likely to participate in the peripheral replication of infectivity and generation of PrP^TSE^ [32]. Consistent with their findings, we detected PrP^TSE^ in spleens as early as 3 mpi (earliest time point tested)—much earlier than in the brain. Considering its probable early involvement in the development of vCJD after peripheral routes of infection, the spleen might be a common site where murine PrP^TSE^ is generated de novo and released into the circulation. Further studies would be needed to confirm this hypothesis. 

Our mouse studies indicated no difference in the times when endogenous PrP^TSE^ was first detected in the blood of mice inoculated with vCJD agent under conditions that mimicked those used to infect cynomolgus and rhesus macaques. This suggested that the reduced inoculum dose used to infect rhesus macaques was unlikely to have caused the delayed appearance of PrP^TSE^ in rhesus blood. We hypothesized that some weak species barrier in rhesus macaques inoculated with cynomolgus-derived inoculum might have allowed vCJD to transmit more efficiently to cynomolgus than to rhesus macaques—an unexpected finding since both species of macaque are genetically very close. Alternatively, natural variability from animal to animal might also explain those differences. In any case, these findings serve as a warning that tests that detect PrP^TSE^ in blood might not always be reliable, reducing their negative predictive value.

In conclusion, independent of the route of experimental exposure to the vCJD agent, PrP^TSE^ was detected in the blood of susceptible mice starting a few months post-exposure and maintained at constant levels in the blood for years. These data support efforts to develop and validate tests for PrP^TSE^ in blood to identify individuals with early TSE infections and, thus, reduce the risk of iatrogenic sCJD. We also detected PrP^TSE^ products amplified from mouse blood by PMCA using a PrP^TSE^ ELISA-like assay that was rapid and reasonably sensitive, with a readout simpler, faster, and less variable than that of Western blots. Improvement of high-throughput ELISA-like assays to detect human PrP^TSE^ might eventually facilitate the development of a human TSE test.

## Figures and Tables

**Table 1:** Summary of inoculation conditions for TgBo110 and PrP-knockout mice.

		Inocula *		
Conditions	Inoculation Routes	IV	IP	IC
cynomolgus condition	IV + IP-high	100 µL 0.4% BH	200 µL 0.4% BH ^#^	-
rhesus condition	IV + IP-low	100 µL 0.4% BH	200 µL 0.04% BH	-
control	IC	-	-	30 µL 1% BH
control	IV	100 µL 0.4% BH	-	-
control	IP-high	-	200 µL 0.4% BH	-
control	IP-low	-	200 µL 0.04% BH	-

* Inoculum abbreviations: IV, intravenous; IP, intraperitoneal; IC, intracerebral. ^#^ BH, brain homogenate.

**Table 2:** Summary of PrP^TSE^ detection for each inoculated mouse.

Inoculation	mpi	Condition *	Blood	Brain	Spleen	Status **
IV + IP-high (cynomolgus conditions)	3	Scheduled euthanasia	Pos	Neg	Pos	Pos
	12	Sick	Pos	Pos	-	Pos
	12	Found dead	No blood	Pos	Pos	Pos
	16	Sick	Pos	Pos	-	Pos
	19	Sick	Pos	Pos	Pos	Pos
	20	Found dead	No blood	Pos	-	Pos
IV + IP-low (rhesus conditions)	4	Scheduled euthanasia	Pos	Neg	-	Pos
	11	Sick	Neg	Neg	No spleen	Neg
	12	Sick	Pos	Neg	-	Pos
	12	Sick	Pos	Neg	-	Pos
	13	Sick	Pos	Neg	-	Pos
	15	Found dead	No blood	Neg	No spleen	Neg
	15	Sick	Pos	Pos	-	Pos
	17	Sick	Pos	Pos	-	Pos
	17	Found dead	No blood	Pos	No spleen	Pos
	18	Sick	Pos	Pos	Neg	Pos
IC	4	Scheduled euthanasia	Pos	Pos	Pos	Pos
	7	Found dead	No blood	Pos	No spleen	Pos
	8	Sick	Pos	Pos	Pos	Pos
	11	Sick	Pos	Pos	-	Pos
	11	Sick	Pos	Pos	-	Pos
	12	Sick	Pos	Pos	-	Pos
	12	Sick	Pos	Pos	-	Pos
	12	Sick	Pos	Pos	-	Pos
IV	4	Scheduled euthanasia	Pos	Neg	Pos	Pos
	11	Sick	Pos	Neg	-	Pos
	15	Found dead	No blood	Pos	Pos	Pos
	16	Sick	Pos	Pos	-	Pos
	17	Found dead	No blood	Pos	No spleen	Pos
	22	Sick	Pos	Pos	Pos	Pos
	22	Sick	Pos	Pos	-	Pos
	24	Sick	Pos	Pos	-	Pos
IP-high	4	Scheduled euthanasia	Pos	Neg	Pos	Pos
	4	Found dead	No blood	Neg	Pos	Pos
	16	Found dead	No blood	Pos	Pos	Pos
	17	Sick	Pos	Pos	-	Pos
	17	Sick	Pos	Pos	-	Pos
	19	Found dead	No blood	Pos	Neg	Pos
	20	Sick	Pos	Pos	-	Pos
IP-low	4	Scheduled euthanasia	Neg	Neg	Neg	Neg
	6	Found dead	No blood	Neg	No spleen	Neg
	17	Sick	Pos	Pos	-	Pos
	22	Sick	Pos	Pos	-	Pos
	22	Sick	Pos	Pos	-	Pos
	22	Sick	Pos	Pos	-	Pos
	22	Sick	Pos	Pos	-	Pos
	23	Sick	Pos	Pos	-	Pos
	24	End of study	Neg	Neg	Neg	Neg
	24	Sick	Pos	Pos	-	Pos

* “Sick” denotes any type of health-related issue, not necessarily vCJD. ** Health status at time of death. “-” indicates not tested.

## Data Availability

Not applicable.

## Supplementary Materials

The following supporting information can be downloaded at: https://www.mdpi.com/article/10.3390/v15071466/s1, Figure S1: Experimental design.
