# Supplementary figures and images for: Kinetics of Abnormal Prion Protein in Blood of Transgenic Mice Experimentally Infected by Multiple Routes with the Agent of Variant Creutzfeldt–Jakob Disease
Source: Viruses. 2023 Jun 28;15(7):1466. doi: 10.3390/v15071466 (PMC10384726; doi:10.3390/v15071466)

Figure S1

## Experimental design

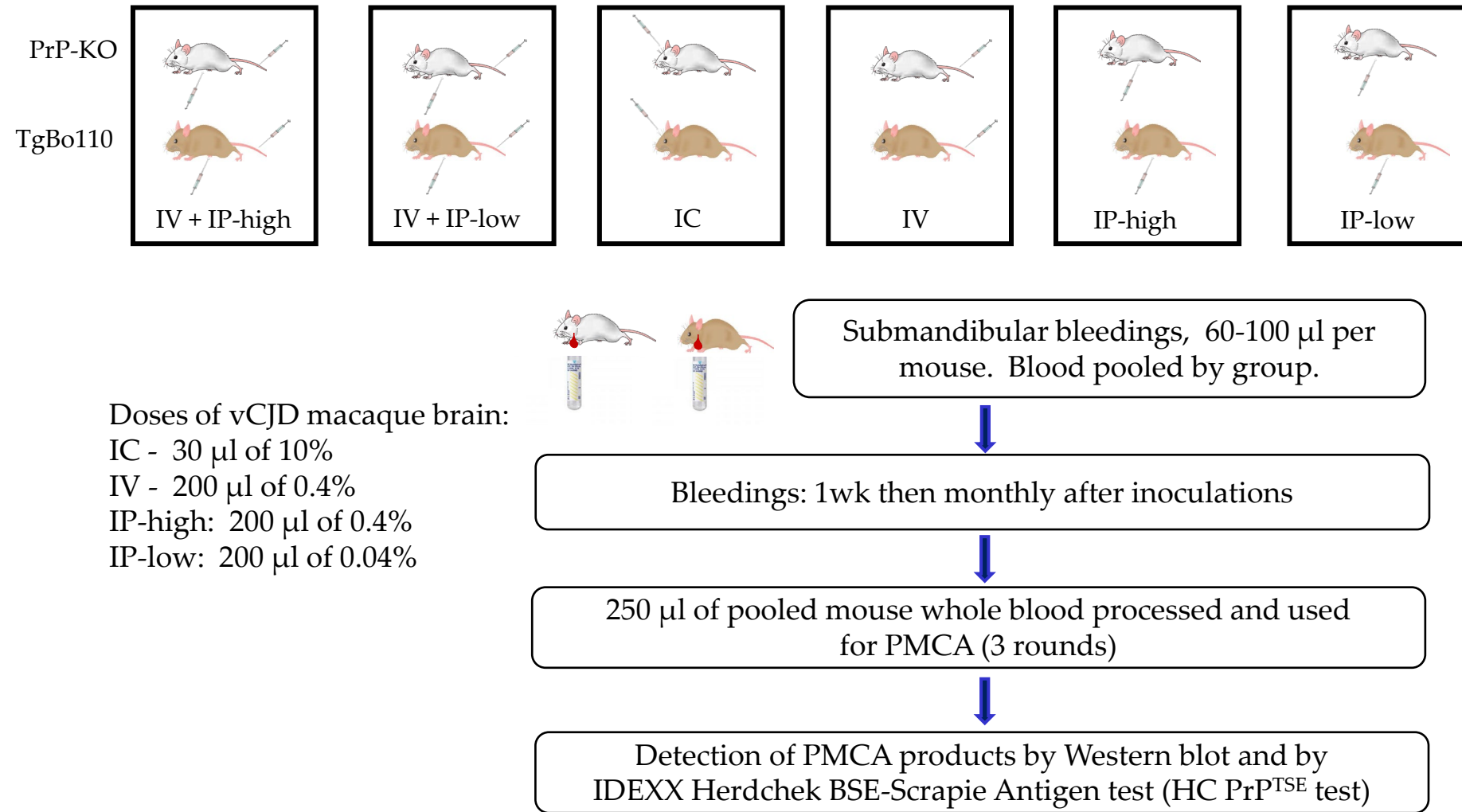

Supplement: Supplementary file 1 [file viruses-15-01466-s001.zip › viruses-2414577-supplementary.pdf]
